# Phenotypic Progression of Stargardt Disease in a Large Consanguineous Tunisian Family Harboring New *ABCA4* Mutations

**DOI:** 10.1155/2018/1030184

**Published:** 2018-03-15

**Authors:** Yousra Falfoul, Imen Habibi, Ahmed Turki, Ahmed Chebil, Asma Hassairi, Daniel F. Schorderet, Leila El Matri

**Affiliations:** ^1^Hedi Rais Institute of Ophthalmology (Department B), Tunis, Tunisia; ^2^Oculogenetic Laboratory LR14SP01, Tunis, Tunisia; ^3^Institute for Research in Ophthalmology (IRO), Sion, Switzerland; ^4^Department of Ophthalmology, University of Lausanne and Faculty of Life Sciences, Ecole Polytechnique Fédérale of Lausanne, Lausanne, Switzerland

## Abstract

To assess the progression of Stargardt (STGD) disease over nine years in two branches of a large consanguineous Tunisian family. Initially, different phenotypes were observed with clinical intra- and interfamilial variations. At presentation, four different retinal phenotypes were observed. In phenotype 1, bull's eye maculopathy and slight alteration of photopic responses in full-field electroretinography were observed in the youngest child. In phenotype 2, macular atrophy and yellow white were observed in two brothers. In phenotype 3, diffuse macular, peripapillary, and peripheral RPE atrophy and hyperfluorescent dots were observed in two sisters. In phenotype 4, Stargardt disease-fundus flavimaculatus phenotype was observed in two cousins with later age of onset. After a progression of 9 years, all seven patients displayed the same phenotype 3 with advanced stage STGD and diffuse atrophy. WES and MLPA identified two *ABCA4* mutations M1: c.[(?_4635)_(5714+?)dup; (?_6148)_(6479_+?) del] and M2: c.[2041C>T], p.[R681^∗^]. In one branch, the three affected patients had M1/M1 causal mutations and in the other branch the two affected patients had M1/M2 causal mutations. After 9-year follow-up, all patients showed the same phenotypic evolution, confirming the progressive nature of the disease. Genetic variations in the two branches made no difference to similar end-stage disease.

## 1. Introduction

The ATP-binding cassette transporter ABCA4, also known as ABCR, is a member of the ATP-binding cassette transporter gene subfamily A [1]. ABCA4 is found in the outer segment disk membrane photoreceptor cells [2] and also marginally present in the brain [3].

ABCA4 is important in the visual cycle, as a retinoid transporter of the toxic all-trans retinal out of the disc for recycling by the retinal pigment epithelium (RPE) [4]. Without this function, an accumulation of toxic all-trans-retinal derivatives in rods and cones may cause an apoptosis of the supporting retinal pigment epithelium cells and, ultimately, degeneration of photoreceptors [5, 6].

Mutations in ABCA4 gene are known to cause Stargardt disease (STGD) which is the most frequent macular dystrophy [7] and typically presents with central macular atrophy and yellow-white dots at the posterior pole, primarily at the level of the RPE. Additional diseases were also linked to mutations in *ABCA4* including fundus flavimaculatus, cone-rod dystrophy (CRD), and retinitis pigmentosa [8–11] or variants considered as a risk factor for age-related macular degeneration [12, 13].

The present study reports the molecular origin and clinical evolution in a large Tunisian family with remarkable variations in STGD and CRD phenotypes. This family was initially published in 2013 [14]. We now present a 9-year follow-up, showing progressive disease in all patients with evolution to a similar phenotype of diffuse macular, peripapillary, and peripheral RPE atrophy and hyperfluorescent dots.

## 2. Patients and Methods

### 2.1. Patients' Recruitment

We followed a large multiplex family originating from Southern Tunisia (Gafsa) whose initial presentation was published previously [12]. Twelve subjects from two branches (A and B) with autosomal recessive STGD disease were enrolled between March 2005 and January 2014. Written informed consent was obtained from each study participant. Analyses were done in accordance with local guidelines, and regulation study was approved by the Local Ethics Committee of the Hedi Rais Institute.

**Figure 1:**
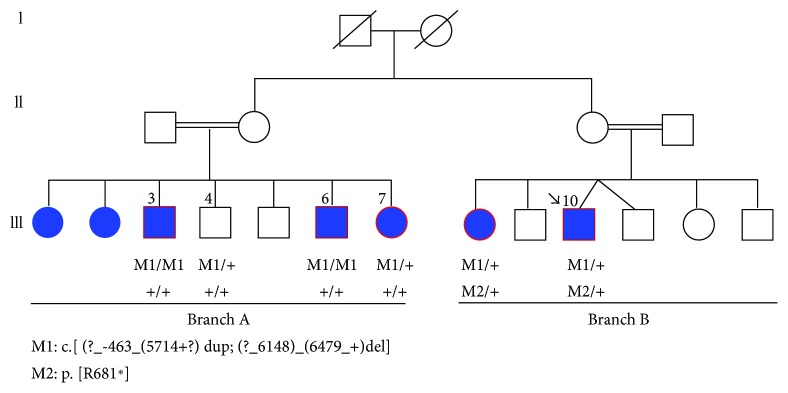
Pedigree structure of the family and segregation analysis of ABCA4 mutations.

In accordance with the Helsinki Declaration, clinical examination was performed in 7 patients, biological material was obtained from 11 members, and family genealogical data were gathered (Figure 1).

### 2.2. Clinical Investigation

A detailed clinical examination including visual acuity, fundus photography, fluorescein angiography, and electroretinography (full-field ERG) were performed for all subjects. The initial data were compared to those at follow-up.

Patients were classified into four phenotypes:

- Phenotype 1: bull's eye maculopathy, slightly altered photopic responses on full-field ERG
- Phenotype 2: macular atrophy and yellow-white dots, altered photopic responses on full-field ERG
- Phenotype 3: diffuse macular, peripapillary, and peripheral RPE atrophy and hyperfluorescent dots, altered photopic and scotopic responses on full-field ERG.
- Phenotype 4: Stargardt disease-fundus flavimaculatus (STGD-FFM) with central atrophy, yellow-white dots, hyperfluorescent atrophic spots, and silent choroid, altered photopic responses on full-field ERG (this phenotype appeared similar to phenotype II but was considered a separate nosology due to older age of onset)

### 2.3. Molecular Analysis

Peripheral blood of all subjects was collected for genomic DNA isolation from leukocytes using the “salting out” standard method. A DNA sample of the index patient was subjected to WES. WES was performed using the Roche NimbleGen version 2 paired-end sample preparation kit and Illumina HiSeq2000 at a mean coverage ×31. Sequence reads were aligned to the human genome reference sequence (build hg19), and variants were identified and annotated using the NextGene software package v.2.3.5. (Softgenetics, State College, PA). To determine the regions of homozygosity from the WES data, we used an in-house developed Excel macro.

### 2.4. In Silico Analysis

All variants were first filtered against several public databases for the minor allele frequency (MAF) < 1%. dbSNP served as a reference to exclude any known frequent variants. We only focused on nonsynonymous variants, variants in splicing sites, and frame-shift coding insertions or deletions. The pathogenicity index for the identified missense mutations was calculated in silico using Sorting Intolerant from Tolerant (SIFT) (http://sift.bii.a-star.edu.sg/) and Polymorphism Phenotyping V2 (PolyPhen-2) (http://genetics.bwh.harvard.edu/pph2/). The PhyloP score and Grantham distances were also recorded to check the nucleotide conservation and change in amino acid physiochemical properties.

### 2.5. Mutation Validation

Variants were confirmed by Sanger sequencing, and segregation analysis was done in the family. The following primers designed to amplify exon 14 of *ABCA4* were used: ABCA4-14F: 5′-TCAACAAACATTTATTCTGCCTCT-3′ and *ABCA4*-14R: 5′-AGCTTCTCCAGATGGTCACG-3′. PCR was realized in a total reaction mixture of 20 *μ*l, containing 20 ng of genomic DNA, 10 pmol of each primer (Eurogentec, Liège, Belgium), and 10 *μ*l of FastStart PCR Master Mix (Roche, Basel, Switzerland). The DNA was denatured at 95°C for 1 minute, prior to 35 cycles of amplification of 1 min at 94°C, 1 min at 58°C, 1 min at 72°C, and the final extension step at 72°C for 10 min using a GeneAmp 9700 thermal cycler (Applied Biosystems, Carlsbad, California, USA). The amplified fragment was sequenced using a BigDye terminator sequencing kit (Perkin Elmer). Sequenced samples were purified using Performa®V3 96-well short plate, according to manufacturer's instructions, loaded in a 3100 XL ABI sequencer (Applied Biosystems) and analyzed using ABI Prisms Navigator Software.

### 2.6. MLPA

We performed multiplex ligation-dependent probe amplification (MLPA) on all family members to confirm a large deletion, suspected through lack of PCR amplification in cousins.

The following 2 kits were used: SALSA P151-B1 and P152-B2 kits where both contained probes for all exons of the *ABCA4* gene. MLPA analysis was performed according to manufacturer's instructions (MRC, Holland, Amsterdam, The Netherlands), and data analysis was carried out by GeneMapper software. The MLPA test was performed twice for confirmation of abnormal changes.

## 3. Results

### 3.1. Clinical Data

At the 9-year follow-up control, all 7 patients displayed disease progression with advanced stage STGD and diffuse RPE atrophy.

In branch A, 5 patients displayed STGD with initially 3 different phenotypes [14]. Patient III7, who originally presented with phenotype 1, developed after 9 years a large central area of pronounced chorioretinal atrophy in both eyes, with yellow-white dots distributed at the posterior pole and midperiphery. The peripapillary area was normal. Visual acuity fell from 20/200 to 20/400. Full-field ERG showed both altered photopic and scotopic responses. Patients III3 and III6 with STGD-FFM phenotype 2, associating macular atrophy, yellow-white dots, hyperfluorescent atrophic spots, and silent choroid, developed diffuse macular, peripapillary, and RPE atrophy extending beyond the vascular arcades. The yellow-white dots in the midperiphery regressed to be replaced by numerous atrophic dots. Full-field ERG showed both altered photopic and scotopic responses. Primary STGD phenotype 3 III1 and III2 patients with diffuse macular, peripapillary, and peripheral RPE atrophy and hyperfluorescent dots progressed to severe CRD, with extensive areas of atrophy, pigment clumping, and migration throughout the posterior pole involving the peripheral retina. Hyperfluorescent atrophic dots progressed with hypofluorescent spots beyond the vascular arcades.

In branch B, 2 patients III8 and III10 presented initial phenotype 4 STGD (Stargardt fundus flavimaculatus, later age of onset) with central atrophy, yellow-white dots, macular atrophy, hyperfluorescent atrophic spots, and silent choroid. After 9-year progression of the disease, both presented CRD with diffuse macular, peripapillary, and peripheral RPE atrophy, regression of the yellow-white dots, and hyperfluorescent dots. Full-field ERG showed severe altered photopic and scotopic responses.

**Figure 2:**
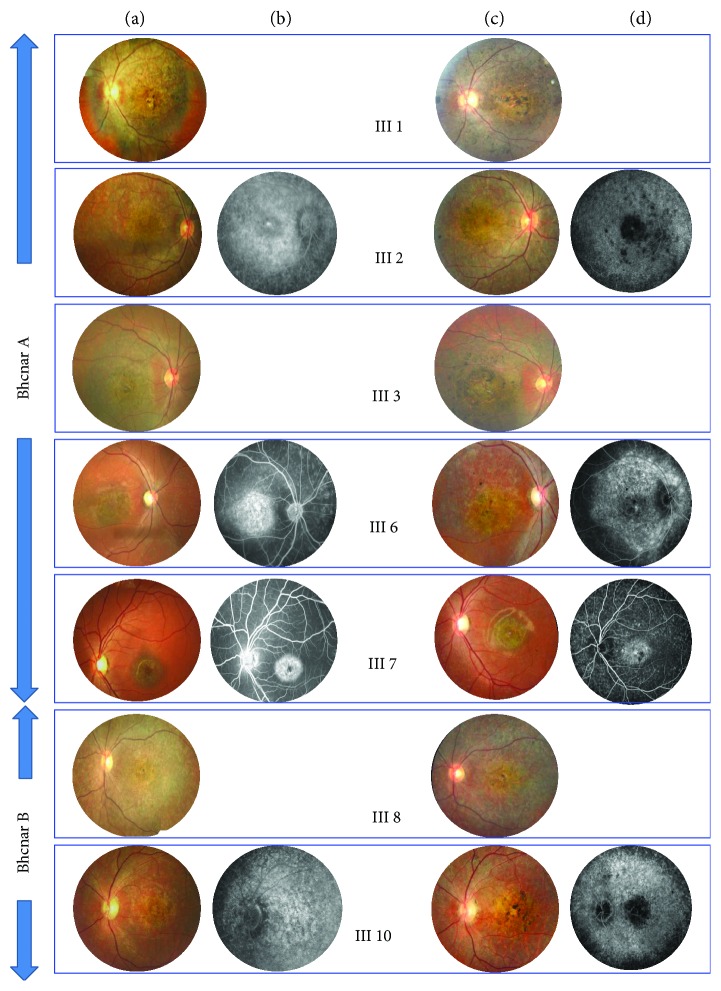
(a, b) Primary phenotype. (c, d) Final phenotype after 9-year progression of the disease.

Phenotype progression of the disease is illustrated in Figure 2.

Clinical results at baseline and follow-up are summarized in Table 1.

### 3.2. Genetic Findings

Homozygosity mapping and Sanger sequencing of retinal dystrophy-associated genes did not reveal any mutation in this consanguineous family.

WES revealed a previously identified nonsense mutation c.[2041C>T]; p.[R681^∗^] found in exon 14, but in heterozygous form in the proband, which did not segregate with the phenotype in the family. The affected sister carried this heterozygous mutation while the three affected cousins were wild type for this variant.

Multiplex ligation-dependent probe amplification was used to search for a potential deletion in the index patient carrying the heterozygous nonsense mutation. This analysis showed a new complex rearrangement in *ABCA4*: a heterozygous deletion of exon 45 to 47 and heterozygous duplication of exon 32 to 40. This rearrangement was also observed in his sister. The index patient was thus a compound heterozygous: c.[2041C>T], p.[R681^∗^]; c.[(?_-4635)_(5714+?)dup; (?_6148)_(6479_+?) del].

The three affected cousins with no previously detected mutation were then tested by MLPA, identifying the similar complex rearrangement in a homozygous state.

## 4. Discussion

The aim of the current study was to provide a comprehensive genetic and clinical profile of ABCA4-associated diseases in a consanguineous Tunisian family. In the index patient of branch A, we identified a new genomic rearrangement c.[(?_-4635)_(5714+?)dup; (?_6148)_(6479_+?) del] in *ABCA4*. This rearrangement was present in a homozygous state in the two affected brothers. To date, large genomic rearrangements account for a minor portion of STGD disease alleles. The first one was described by Yatsenko et al. [15]. In our cohort, this is the first large genomic rearrangement identified in Tunisian STGD patients.

It is likely that skipping of exons 45–47 and the duplication of exons 32–40 affect the three-dimensional structure and thereby the function of the protein. Theoretically, these mutations could cause a shift in the reading frame, resulting in a premature termination codon at the NBD2 domain and the absence of C-terminal segment that is crucial for the proper folding of ABCA4 into its native form. Alternatively, nonsense-mediated mRNA decay could inhibit protein production and generate a severe loss of function. A malfunctioning ABCA4 cannot remove N-retinylidene-phosphatidylethanolamine from photoreceptors. As a result, N-retinylidene-PE combines with another substance to produce lipofuscin, which builds up in retinal cells. The buildup of lipofuscin is toxic and causes progressive vision loss in subjects with STGD macular degeneration.

However, in branch B of the family, the two cousins were heterozygous for this large rearrangement and carry a heterozygous nonsense mutation p.[R681^∗^]. This mutation is localized in the transmembrane segment and was described previously by two groups [16, 17]. More than 640 mutations in *ABCA4* have been found in different forms of STGD. It is unclear, however, how mutations in *ABCA4* can cause different ocular disorders. A recent study evaluated the severity of specific ABCA4 alleles and showed that variants localized in the transmembrane segment must produce a sufficient amount of functional ABCA4, but induce deterioration of peripheral retinal function with time [18].

The identification of these two new alleles c.[(?_4635)_(5714+?)dup; (?_6148)_(6479_+?) del] and c.[2041C>T],[(?_4635)_(5714+?)dup; (?_6148)_(6479_+?) del] in a Tunisian family confirms the potential complex variations in *ABCA4*. The segregation analysis was important to confirm the molecular diagnosis. Our analysis could explain the clinical data with different phenotypes and age of onset [14]. Regarding phenotype and severity of visual symptoms, the family showed Stargardt disease at various stages of progression. After a 9-year follow-up, all patients confirmed the progressive nature of the disease. Progressive retinal degeneration, including development/resorption of flecks, atrophy enlargement, and deterioration of retinal function, has been reported in STGD [19, 20].

In patient III7 who initially presented phenotype 1, the loss of visual acuity was accompanied by appearance of yellow-white lesions at the level of RPE, which are referred to as fundus flecks. In full-field ERG, we also found a progression to scotopic and photopic response alterations. Patients with phenotypes 2 and 3 demonstrated atrophic-appearing lesions within the macula and peripheral retina as previously reported with a final severe CRD phenotype as shown in full-field ERG responses [19]. Patients from branch B with primary phenotype STGD-FFM characterized by a later age of onset and a better primary visual acuity progressed also into severe phenotype 3 with severe macular and peripheral atrophy.

In terms of retinal function, STGD has been classified into three full-field ERG groups: group I with normal rod and cone responses; group II with relative loss of photopic function; and group III with both altered photopic and scotopic functions [11]. ERG findings in our two branches with primary different ERG responses from preserved photoreceptor response to both photopic and scotopic altered responses and a final severely altered ERG responses highlighted the assumption of different stages of progression course of the disease from macular dystrophy to CRD in ABCA4 mutations.

It has been demonstrated that genetic variability influenced the primary severity of the condition and that age of onset correlated with the amount of ABCR activity in photoreceptors [21, 22]. In addition, it was recognized that STGD-FFM had an older age of onset as observed in our patients and had slower progression and thus better prognosis [23]. However, in our two STGD-FFM patients, progression was towards severe diffuse macular, peripapillary, and peripheral RPE atrophy and regression of the yellow-white dots joining severe phenotype 3 shown in branch A, irrespective of the haplotype variability. This would suggest that the primary phenotype observed corresponds to a stage of progression of the disease in both branches and that genotypic difference between M1/M1 versus M1/M2 will influence age of onset, degree of progression, and rapidity of development of the final phenotype less severe in branch B.

In conclusion, the observed progression of disease supports the hypothesis that all phenotypes described at an initial stage of disease are variable stages of a single progressive disease, depending on age in family A and on various haplotype combinations in family B.

We report two genetically different abnormalities in a STGD consanguineous family. The mechanism by which the large homozygous rearrangement, or the compound heterozygous mutations, leads to the same phenotype after 9 years of follow-up is not fully understood. Further biochemical and functional studies should provide deeper insights into the pathogenesis of STGD.

## Figures and Tables

**Table 1:** Clinical results at baseline and follow-up: visual acuity, fundus appearance, and full-field ERG responses.

Family	Code sex	Age of onset	Primary visual acuity	Fundus and FA at baseline	Primary full-field ERG	Baseline diagnosis	Final visual acuity	Fundus and FA at last follow-up	Final full-field ERG	Last follow-up diagnosis
Family A	III1F	6	<20/400	Diffuse macular, peripapillary, and peripheral RPE atrophy; hyperfluorescent dots	Altered photopic and scotopic responses	Stargardt dystrophy “phenotype III”	Light perception	Extensive areas of atrophy, pigment clumping, and migration throughout the posterior pole, to the peripheral retina; hyperfluorescent atrophic dots progression with hypofluorescent spots beyond the vascular arcades	Altered photopic and scotopic responses	Severe Stargardt dystrophy “phenotype III”
	III2F	6	<20/400	Diffuse macular, peripapillary, and peripheral RPE atrophy; hyperfluorescent dots	Altered photopic and scotopic responses	Stargardt dystrophy “phenotype III”	Light perception	Extensive areas of atrophy, pigment clumping, and migration throughout the posterior pole, to the peripheral retina	Altered photopic and scotopic responses	Severe Stargardt dystrophy “phenotype III”
	III3M	6	20/330	Macular atrophy; white-yellow flecks; hyperfluorescent atrophic spots; silent choroid	Altered photopic responses	Stargardt fundus flavimaculatus “phenotype II”	Hand movement	Diffuse macular, peripapillary, and RPE atrophy extending beyond the vascular arcades	Altered photopic and scotopic responses	Stargardt dystrophy “phenotype III”
	III6M	6	20/400	Central atrophy with white-yellow flecks; macular atrophy; hyperfluorescent atrophic spots; silent choroid	Altered photopic responses	Stargardt fundus flavimaculatus “phenotype II”	Light perception	Diffuse macular, peripapillary, and RPE atrophy extending beyond the vascular arcades; regression of the white-yellow flecks in the midperiphery replaced by numerous atrophic dots	Altered photopic and scotopic responses	Stargardt dystrophy “phenotype III”
	III7F	5	20/200	Bull's eye maculopathy; temporal peripapillary atrophy; silent choroid; fibroglial scar	Slightly altered photopic responses	Stargardt maculopathy “phenotype I”	20/400	Central atrophy with white-yellow flecks; macular atrophy; hyperfluorescent atrophic spots; silent choroid	Altered photopic and scotopic responses	Stargardt fundus flavimaculatus “phenotype III”

Family B	III8F	10	20/500	Central atrophy with white-yellow flecks; hyperfluorescent atrophic spots; silent choroid	Altered photopic responses	Stargardt fundus flavimaculatus	Light perception	Diffuse macular, peripapillary, and peripheral RPE atrophy; regression of the white-yellow flecks hyperfluorescent dots	Altered photopic and scotopic responses	Stargardt dystrophy “phenotype III”
	III10M	12	20/400	Central atrophy with white-yellow flecks; macular atrophy hyperfluorescent atrophic spots; silent choroid	Altered photopic responses	Stargardt fundus flavimaculatus	Light perception	Diffuse macular, peripapillary, and peripheral RPE atrophy; regression of the white-yellow flecks hyperfluorescent dots	Altered photopic and scotopic responses	Stargardt dystrophy “phenotype III”
